# Supplementary material for: Resolving the full spectrum of human genome variation using Linked-Reads
Source: Genome Res. 2019 Apr;29(4):635–45. doi: 10.1101/gr.234443.118 (PMC6442396; doi:10.1101/gr.234443.118)
Supplement: Supplemental Material [file supp_gr.234443.118_Supplemental_longranger-2.2.zip › longranger-2.2.tar.bz2!/lr2.2-public/lib/stan/cmdstan/stan/doc/api/html/rev_2functions_2hypot_8hpp_source.html]

Stan: src/stan/agrad/rev/functions/hypot.hpp Source File


|  |  |
| --- | --- |
|  | Stan  2.5.0  probability, sampling & optimization |


- Main Page
- Related Pages
- Namespaces
- Classes
- Files

- File List
- File Members

All Classes Namespaces Files Functions Variables Typedefs Enumerations Enumerator Friends Macros Pages

- src
- stan
- agrad
- rev
- functions

hypot.hpp

Go to the documentation of this file.

1 #ifndef STAN\_\_AGRAD\_\_REV\_\_FUNCTIONS\_\_HYPOT\_HPP

2 #define STAN\_\_AGRAD\_\_REV\_\_FUNCTIONS\_\_HYPOT\_HPP

3

4 #include <valarray>

5 #include <stan/agrad/rev/var.hpp>

6 #include <stan/agrad/rev/internal/vv\_vari.hpp>

7 #include <stan/agrad/rev/internal/v\_vari.hpp>

8 #include <math.h>

9

10 namespace stan {

11  namespace agrad {

12

13  namespace {

14  class hypot\_vv\_vari : public op\_vv\_vari {

15  public:

16  hypot\_vv\_vari(vari\* avi, vari\* bvi) :

17  op\_vv\_vari(::hypot(avi->val\_,bvi->val\_),

18  avi,bvi) {

19  }

20  void chain() {

21  avi\_->adj\_ += adj\_ \* avi\_->val\_ / val\_;

22  bvi\_->adj\_ += adj\_ \* bvi\_->val\_ / val\_;

23  }

24  };

25

26  class hypot\_vd\_vari : public op\_v\_vari {

27  public:

28  hypot\_vd\_vari(vari\* avi, double b) :

29  op\_v\_vari(::hypot(avi->val\_,b),

30  avi) {

31  }

32  void chain() {

33  avi\_->adj\_ += adj\_ \* avi\_->val\_ / val\_;

34  }

35  };

36  }

37

54  inline var hypot(const stan::agrad::var& a,

55  const stan::agrad::var& b) {

56  return var(new hypot\_vv\_vari(a.vi\_,b.vi\_));

57  }

58

73  inline var hypot(const stan::agrad::var& a,

74  const double& b) {

75  return var(new hypot\_vd\_vari(a.vi\_,b));

76  }

77

119  inline var hypot(const double& a,

120  const stan::agrad::var& b) {

121  return var(new hypot\_vd\_vari(b.vi\_,a));

122  }

123

124  }

125 }

126 #endif

stan::agrad::hypot

fvar< T > hypot(const fvar< T > &x1, const fvar< T > &x2)

**Definition:** hypot.hpp:16

stan::agrad::var::vi\_

vari \* vi\_

Pointer to the implementation of this variable.

**Definition:** var.hpp:40

v\_vari.hpp

var.hpp

stan::agrad::var

Independent (input) and dependent (output) variables for gradients.

**Definition:** var.hpp:27

vv\_vari.hpp


---

[ Stan Home Page ]

*© 2011–2014,
Stan Development Team.*
